# Large multimodality model fine-tuned for detecting breast and esophageal carcinomas on CT: a preliminary study

**DOI:** 10.1007/s11604-024-01718-w

**Published:** 2024-12-13

**Authors:** Koichiro Yasaka, Motohide Kawamura, Yuki Sonoda, Takatoshi Kubo, Shigeru Kiryu, Osamu Abe

**Affiliations:** 1 Department of Radiology, Graduate School of Medicine, The University of Tokyo, 7-3-1 Hongo, Bunkyo-Ku, Tokyo, 113-8655 Japan; 2 Department of Radiology, International University of Health and Welfare Narita Hospital, 852 Hatakeda, Narita, Chiba 286-0124 Japan

**Keywords:** Large multimodality model, Esophageal carcinoma, Breast carcinoma, Computed tomography, Deep learning

## Abstract

**Purpose:**

This study aimed to develop a large multimodality model (LMM) that can detect breast and esophageal carcinomas on chest contrast-enhanced CT.

**Materials and methods:**

In this retrospective study, CT images of 401 (age, 62.9 ± 12.9 years; 169 males), 51 (age, 65.5 ± 11.6 years; 23 males), and 120 (age, 64.6 ± 14.2 years; 60 males) patients were used in the training, validation, and test phases. The numbers of CT images with breast carcinoma, esophageal carcinoma, and no lesion were 927, 2180, and 2087; 80, 233, and 270; and 184, 246, and 6919 for the training, validation, and test datasets, respectively. The LMM was fine-tuned using CT images as input and text data (“suspicious of breast carcinoma”/ “suspicious of esophageal carcinoma”/ “no lesion”) as reference data on a desktop computer equipped with a single graphic processing unit. Because of the random nature of the training process, supervised learning was performed 10 times. The performance of the best performing model on the validation dataset was further tested using the time-independent test dataset. The detection performance was evaluated by calculating the area under the receiver operating characteristic curve (AUC).

**Results:**

The sensitivities of the fine-tuned LMM for detecting breast and esophageal carcinomas in the test dataset were 0.929 and 0.951, respectively. The diagnostic performance of the fine-tuned LMM for detecting breast and esophageal carcinomas was high, with AUCs of 0.890 (95%CI 0.871–0.909) and 0.880 (95%CI 0.865–0.894), respectively.

**Conclusions:**

The fine-tuned LMM could detect both breast and esophageal carcinomas on chest contrast-enhanced CT with high diagnostic performance.

**Secondary abstract:**

Usefulness of large multimodality models in chest cancer imaging has not been assessed so far. The fine-tuned large multimodality model could detect breast and esophageal carcinomas with high diagnostic performance (area under the receiver operating characteristic curve of 0.890 and 0.880, respectively).

## Introduction

Breast and esophageal carcinomas are common carcinomatous types worldwide. One in 8–10 women suffers from breast carcinoma [1]. Esophageal carcinoma is the ninth most common type of carcinoma [2]. Although CT is not a screening modality for detecting these carcinomas, they are not so rare that they are found on CT incidentally because of the increase in the number of CT examinations [3]. Precise diagnosis of breast and esophageal carcinomas on CT can facilitate earlier therapeutic intervention.

Since the mid-2010s, the number of studies on the application of deep learning in radiology has been increasing [4–8]. Deep learning can be applied to detecting [9–12], staging [13–17], and classifying [18–20] lesions. Despite their merits, most deep learning models have been developed for a single task, and this feature of conventional deep learning models can be represented as the word of “weak artificial intelligence.” Most recently, large language models or large multimodality models (LMMs) have gained social attention. Although most conventional deep learning models developed for diagnosing diseases output scalar or vector data, LMMs can return the output data in the form of natural language. The most well-known LMM is GPT-4, which is available from Open AI. Applications of this LMM to radiological tasks have been attempted [21–23]. For example, GPT-4 has been reported to be able to generate radiological reports [24]. However, the use of this model requires external data transmission; thus, privacy protection must be ensured during this process [25]. This hinders the application of GPT-4 to large-scale data. In contrast, other LMMs can be downloaded to local computers. Although LMMs comprise a large number of parameters, dedicated techniques (e.g., parameter-efficient fine-tuning technique) allow the efficient fine-tuning of these models. Considering these factors, we hypothesized that LMMs can be fine-tuned to perform radiological tasks on a local computer and that fine-tuned LMMs can detect both breast and esophageal carcinomas on CT images.

This study assessed whether fine-tuning LMMs is possible on a local desktop computer equipped with a single graphic processing unit and whether fine-tuned LMMs can successfully detect breast and esophageal carcinomas on chest contrast-enhanced CT.

## Materials and methods

This retrospective study was approved by our Institutional Review Board, which waived the requirement for written informed consent considering the retrospective nature of this study.

### Patients

**Fig. 1:**
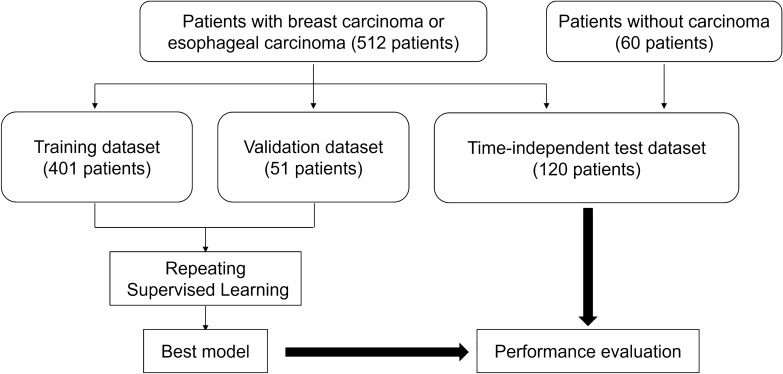
Flowchart of the patient selection process

In this study, CT images of patients who underwent contrast-enhanced CT examination that included the chest region and were included in previous studies [9, 11] were used (512 patients were overlapped). The structure of the model was different between the previous studies [9, 11] (conventional convolutional neural network [26]) and this study (state-of-the-art vision and language model/LMM [bootstrapping language-image pre-training with frozen image encoders and large language models: BLIP-2] [27]). CT images were allotted to the training, validation, and test datasets based on the date of the CT examination, aiming to evaluate the performance of the developed model on a time-independent test dataset (Fig. 1).

### CT imaging

Contrast-enhanced CT examination was performed using CT scanners from two vendors (Canon Medical Systems [Otawara, Japan] and GE Healthcare [Waukesha, WI]). A contrast-enhancement material was injected via the peripheral vein within 60 s, and the scan was initiated 90 s after starting the injection. The scanning and reconstruction parameters for Canon-CT/GE-CT were as follows: tube current, automatic tube current modulation with a standard deviation/noise index of 13.0/11.36; tube voltage, 120 kVp for both; helical pitch, 0.8125/0.984; gantry rotation time, 0.5 s for both; slice thickness, 5 mm for both; and slice interval, 5 mm for both.

### Reference data: text data on diagnosis

For the training and validation datasets, a radiologist with 14 years of imaging experience reviewed all CT images slice by slice and recorded the presence of breast and esophageal carcinomas in a csv file. For slices in which breast and esophageal carcinomas were present, the phrases “suspicious of breast carcinoma” and “suspicious of esophageal carcinoma”, respectively, were recorded. For slices where breast and esophageal carcinomas are absent, the phrase “no lesion” was recorded. In these processes, the histopathological records and, if present, mammography and ultrasound records for the breast carcinoma and endoscopy records for the esophageal carcinoma were referenced. For the test dataset, another radiologist with 11 years of imaging experience was also involved in establishing the reference standard, and the evaluation was performed by consensus reading.

### Data preprocessing

CT images obtained in regions other than the chest region were excluded from the analysis. Furthermore, because there was an imbalance in the numbers of CT slices in which carcinomas were present and absent, CT slices without lesions were under-sampled, and the number of these CT slices was reduced to 1/10 for the training and validation datasets. Using Pydicom (https://pydicom.github.io/), CT images with digital imaging and communications in the medicine format were rescaled so that their appearance was the same as the soft tissue window setting and were saved in the jpg format (512 × 512 pixels).

### Implementation and fine-tuning of the LMM

The LMM was fine-tuned on a computer equipped with a graphics processing unit of Quadro P5000 (NVIDIA), a central processing unit of Core(TM) i9-9900 K (Intel), and 64.0 GB of random-access memory. PyTorch (version 2.1.1; https://pytorch.org/) and Transformers (version 4.35.2; https://huggingface.co/) were used.

The image and text data were processed using the AutoProcessor function (https://huggingface.co/Salesforce/blip2-opt-2.7b). This processor comprises BlipImageProcessor, an image processor, and GPT2TokenizerFast, a tokenizer, with a vocabulary size of 50,265.

**Fig. 2:**
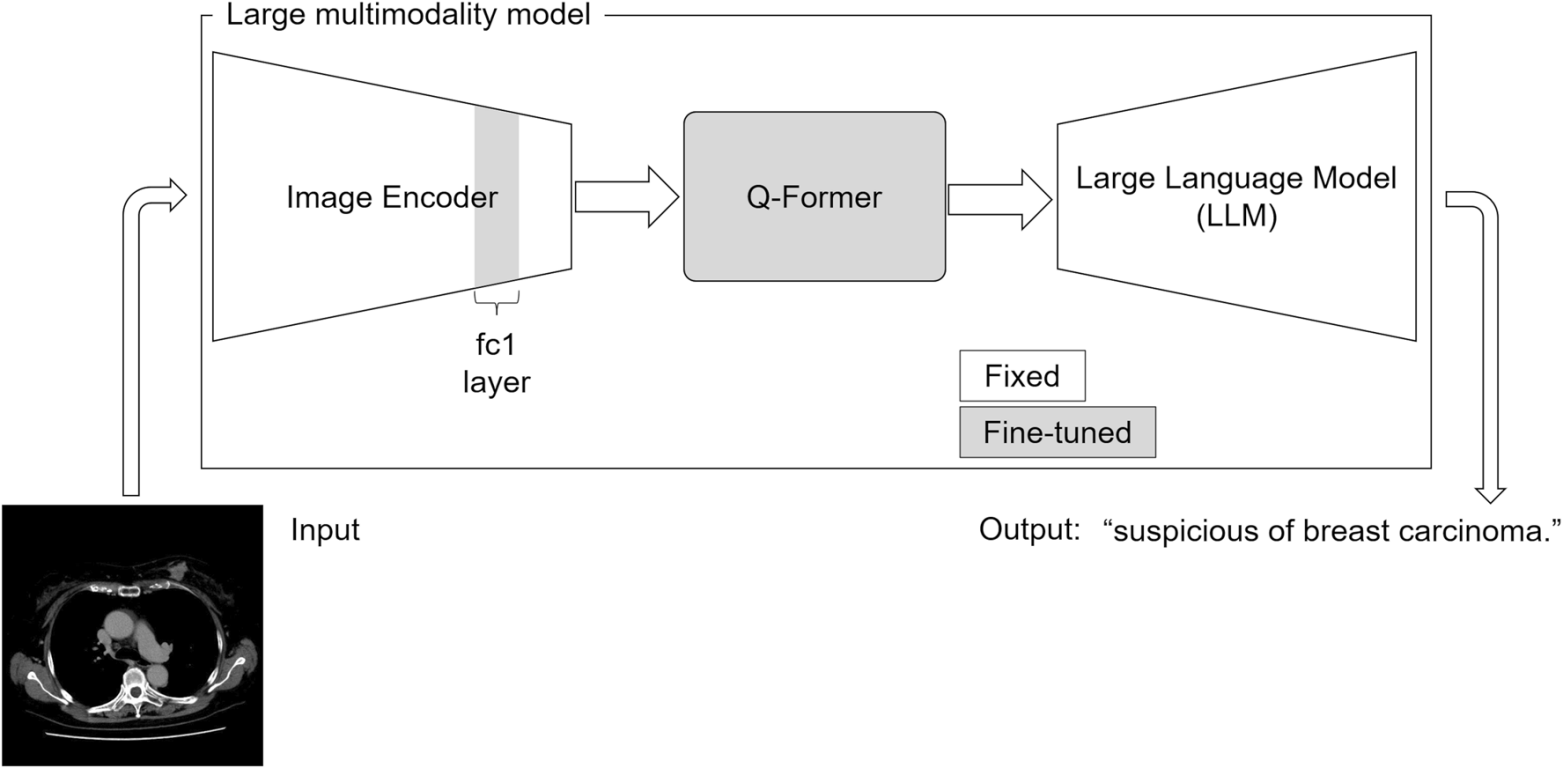
Conceptual image of the fine-tuning of the large multimodality model

BLIP-2 (https://huggingface.co/Salesforce/blip2-opt-2.7b) [27], which is a pre-trained vision and language model, was fine-tuned. The model comprised the vision, q-former, and language models (Fig. 2), and the parameters in the fc1 layer in the vision and q-former (query, key, and dense layers) models were fine-tuned using Low-Rank-Adaptation (r = 16, lora_alpha = 32, lora_dropout = 0.3, bias = “lora_only”) [28]. The other hyperparameters were as follows: epoch, 3; and optimizer, Adam with lr = 5e-4. These hyperparameters were tuned using only the training and validation datasets. Because of the random nature of the training process, supervised learning was performed 10 times (10 trials), and the best performing model on the validation dataset was selected. The performance of the best performing model was further tested using the time-independent test dataset. The code used for fine-tuning the LMM can be made available upon reasonable request.

### Reader test

To compare the performance of the fine-tuned model with readers, two radiology residents (reader A and B with imaging experience of 2 years and 0 year, respectively) were involved. They independently evaluated each image for the presence of malignant lesions. They were blinded to patient background information. Before the reader test, the radiologist with 14 years of imaging experience randomized all image datasets.

### Statistical analysis

All statistical analyses were performed using R (version 4.1.2; https://www.r-project.org/). The model’s output was judged as correct when it exactly matched the reference data (“suspicious of breast carcinoma”/”suspicious of esophageal carcinoma”/”no lesion”). Receiver operating characteristic (ROC) curve analysis was performed to calculate the area under the ROC curve (AUC) for the performance of the LMM and readers in detecting carcinomas. AUC of the LMM was compared with those of readers with the DeLong test. Because of the multiple comparisons (LMM *vs.* reader 1 and LMM *vs.* reader 2), the Bonferroni correction was performed. A *p*-value of less than 0.025 (= 0.050 / 2) was considered to indicate a statistically significant difference.

## Results

### Patients

In the training, validation, and test datasets, 201, 26, and 30 patients with breast carcinoma and 200, 25, and 30 patients with esophageal carcinoma, respectively, were included in this study. Patients without lesions (*n* = 60) were included in the test datasets. In total, 401, 51, and 120 patients were included in the training (age, 62.9 ± 12.9 years; 169 males and 232 females; 5,194 images), validation (age, 65.5 ± 11.6 years; 23 males and 28 females; 583 images), and test (age, 64.6 ± 14.2 years; 60 males and 60 females; 7,349 images) datasets, respectively. The numbers of CT images for the breast carcinoma, esophageal carcinoma, and no lesion categories used in the final analyses were 927, 2180, and 2087; 80, 233, and 270; and 184, 246, and 6919 for the training, validation, and test datasets, respectively. Note that because under-sampling was performed for the no lesion category in the training and validation datasets, the proportion for the number of patients in this category is relatively low compared with that in the test dataset.

The mean sizes of breast carcinomas on the training, validation, and test datasets were 31.6, 32.0, and 37.8 mm, respectively. The mean lengths of esophageal carcinomas in the training, validation, and test datasets were 54.6, 46.6, and 41.2 mm, respectively. T stages (Tis and T1/T2/T3/T4) for the breast carcinoma were the following: 76/87/24/14, 15/8/1/2, and 16/11/2/1 for the training, validation, and test dataset, respectively. T stages for the esophageal carcinoma were the following: 27/72/81/20, 4/8/11/2, and 3/17/9/1, for the training, validation, and test dataset, respectively.

### Performance of the LMM and readers

**Fig. 3:**
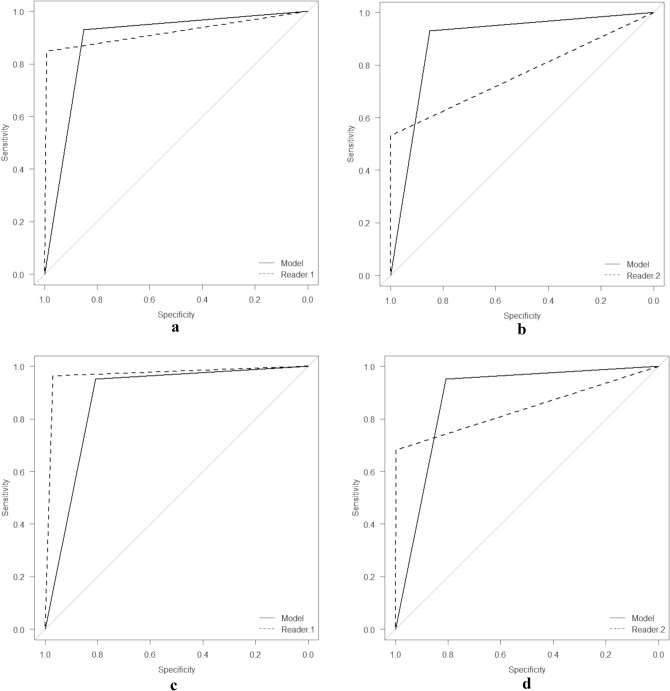
Receiver operating characteristic curves for detecting breast carcinoma (**a, b**) and esophageal carcinoma (**c, d**) by the fine-tuned large multimodality model (**a–d** [solid line]), reader 1 (**a, c** [dotted line]) and reader 2 (**b, d** [dotted line]) in the test dataset

**Fig. 4:**
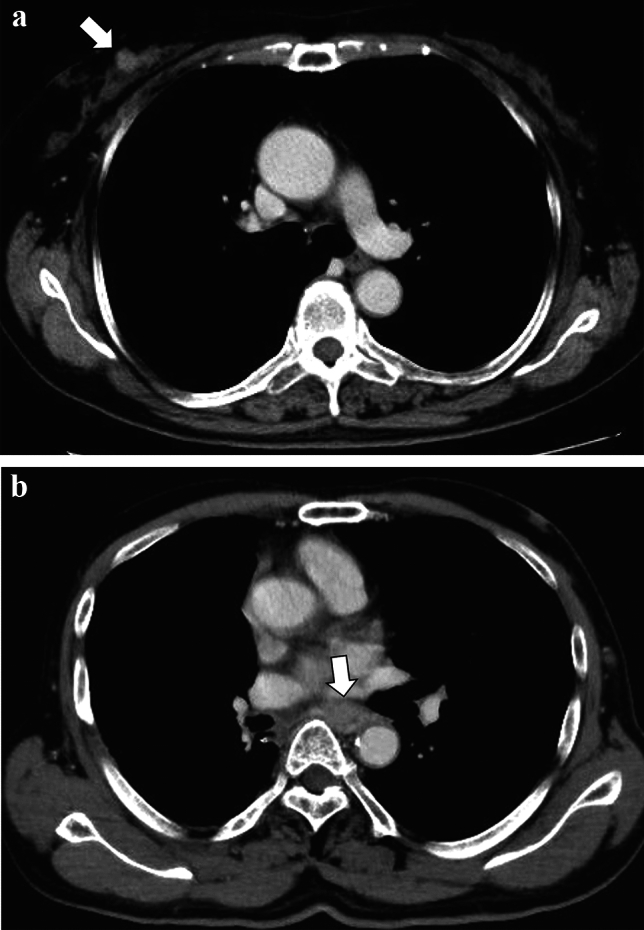
CT images of a 70-year-old female patient with a right breast carcinoma (**a**) and a 78-year-old male patient with esophageal carcinoma (**b**). Both carcinomas (white arrows in (**a**) and (**b**)) were detected by the fine-tuned large multimodality model and reader 1, while reader 2 missed those lesions

The macroaverage sensitivity and accuracy of the fine-tuned LMM that performed best among the 10 trials were 0.761 and 0.768, respectively (Table 1). The performance of this model (trial 4) was further evaluated on the test dataset. The sensitivities of the fine-tuned LMM in the test dataset for detecting breast and esophageal carcinomas were 0.929 and 0.951, respectively, which were significantly higher than those of readers (0.533–0.847 and 0.683–0.963, respectively), except for the sensitivity of model *vs.* reader 1 in esophageal carcinoma (Table 2). The diagnostic performance of the fine-tuned LMM for detecting breast and esophageal carcinomas was high, with AUC values of 0.890 (95%CI 0.871–0.909) and 0.880 (95%CI 0.865–0.894), respectively (Fig. 3). Though the diagnostic performance of the fine-tuned LMM in detecting breast and esophageal carcinomas was lower than that of reader 1 (0.920 [95%CI 0.894–0.946] [*p* = 0.025] and 0.967 [95%CI 0.955–0.979] [*p* < 0.001], respectively), it performed significantly better than reader 2 (0.766 [95%CI 0.730–0.802] [*p* < 0.001] and 0.840 [95%CI 0.811–0.869] [*p* = 0.012], respectively) (Fig. 4).

**Table 1:** Image-based performance of the model in the validation dataset

Trial	Sensitivity				Accuracy
	Breast carcinoma	Esophageal carcinoma	No lesion	Macroaverage	
1	0.225	0.682	0.941	0.616	0.739
2	0.013	0.391	0.578	0.327	0.425
3	0.400	0.807	0.822	0.676	0.758
**4**	**0.713**	**0.897**	**0.674**	**0.761**	**0.768**
5	0.250	0.927	0.719	0.632	0.738
6	0.863	0.936	0.374	0.724	0.666
7	0.400	0.777	0.852	0.676	0.760
8	0.650	0.948	0.552	0.717	0.724
9	0.013	0.549	0.730	0.430	0.559
10	0.775	0.850	0.593	0.739	0.720

The best performing model in the average sensitivity and accuracy is highlighted with bold

**Table 2:** The confusion matrix of the fine-tuned large multimodality model and readers in the test dataset

	Output			Sensitivity	
Reference standard	Breast carcinoma	Esophageal carcinoma	No lesion	Values	Comparison (*vs.* model)
	Model				
Breast carcinoma (184 images)	171	1	12	0.929	
Esophageal carcinoma (246 images)	2	234	10	0.951	
No lesion (6919 images)	1070	1363	4486	0.648	
	Reader 1				
Breast carcinoma (184 images)	156	0	28	0.847	0.005*
Esophageal carcinoma (246 images)	0	237	9	0.963	0.663
No lesion (6919 images)	57	213	6649	0.961	< 0.001*
	Reader 2				
Breast carcinoma (184 images)	98	0	86	0.533	< 0.001*
Esophageal carcinoma (246 images)	0	168	78	0.683	< 0.001*
No lesion (6919 images)	0	19	6900	0.997	< 0.001*

Comparison of sensitivity was performed with the McNemar test

^*^indicate a statistically significant difference

## Discussion

From this preliminary study, we found that LMMs can be fine-tuned on a local desktop computer equipped with a graphics processing unit and that the fine-tuned model could detect both breast and esophageal carcinomas on chest contrast-enhanced CT with high diagnostic performance significantly better than a less-experienced reader.

Studies have reported that breast carcinoma could be detected automatically using deep learning models [9, 10]. Furthermore, deep learning models with the ability to detect esophageal carcinomas have been developed [11, 12]. However, the deep learning models developed in these studies could detect a single type of carcinomas. The merit of the fine-tuned LMM developed in our study lies in its ability to detect both breast and esophageal carcinomas simultaneously with high performance. Because the reference data can be provided in natural language as we have done in our study (“suspicious of breast carcinoma,” “suspicious of esophageal carcinoma,” and “no lesion”), LMMs may have the potential to detect several other types of tumors when trained with data on those tumors.

BLIP-2, which we used in this study, is one of the LMMs. This model comprises the image encoder, q-former, and large language models. Q-former is a lightweight transformer that feeds visual features to the large language model to output the desired text [27]. For fine-tuning this model, we used Low-Rank-Adaptation, which is one of the parameter-efficient fine-tuning techniques. This freezes the pre-trained model weights and injects trainable rank decomposition matrices into the model layers, reducing the number of parameters that should be fine-tuned [28]. This would have allowed the fine-tuning of the LMM on a desktop computer with a single graphics processing unit in the current task. For our study, we fine-tuned the parameters in the fc1 layer of the image encoder in addition to the query, key, and dense layers of the q-former model. This resulted from the fact that adding the fc1 layer of the image encoder for fine-tuning resulted in better performance in our preliminary assessment using the training and validation datasets. In contrast, the large language model in the LMM was not fine-tuned.

This study has some limitations. First, although large models tend to require large numbers of dataset for training, the number of images included in the training dataset in our study was relatively small (5194 images). In addition, it is known from existing research that single-task-specific models often achieve better performance than multitasking models. These may have been the reasons for the relatively lower diagnostic performance in detecting breast carcinoma (AUC = 0.890) and esophageal carcinoma (AUC = 0.880) than previous reports (AUC = 0.967 and 0.910–0.950 for breast carcinoma [9] and esophageal carcinoma [11, 12], respectively). Future studies including larger numbers of patients are necessary to consolidate the results of our study. Second, the model’s performance was not externally validated using geographic sets. According to Walston, et al., temporal and geographic sets are both categorized as external datasets, while random splitting, cross-validation, and leave-one-out methods fall into internal datasets [29]. In our study, to enhance the robustness of results as much as possible, a time-independent test dataset was used. Third, there was a relatively wide variance in the sensitivity of the models among the 10 trials (particularly that for breast carcinoma [0.013–0.863]), indicating instability in the fine-tuning process of the LMM. Fourth, benign breast lesions, such as fibroadenoma, phyllodes tumors, cysts, and mastopathy, were not included in this study. Because of this, there can be risks of overlooking of those benign lesions and misclassification of those lesions as malignant. However, overlooking of benign lesions would not be clinically problematic. In addition, other modalities, such as ultrasonography and mammography, would be more effective in the differential diagnosis of breast lesions rather than CT. Fifth, the developed model only answers whether breast or esophageal cancer is present. Developing the model which provides more detailed information, such as the exact location of the cancer (specific slice and area) and its stage, requires further ingenuity. Future studies focusing on this issue by including large numbers of diseases with various locations would be needed. Finally, carcinomas arising from organs other than the breast and esophagus cannot be detected by our model. The reasons for focusing on those carcinomas in this study were the following: it is common to detect lung carcinomas and bone tumors with CT images set at the lung and bone window settings, respectively. As for the mediastinal tumors, the incidence is much lower than that of breast carcinoma and esophageal carcinoma. While thyroid nodules are detected commonly at CT examination, most of them are benign. Future studies including carcinomas arising from several other organs are expected.

In conclusion, fine-tuned LMMs could detect both breast and esophageal carcinomas simultaneously with high diagnostic performance significantly better than a less-experienced reader. Because reference data can be provided in a natural language format for fine-tuning the LMM, carcinomas arising from several other organs may be detected using a single model. Future research into developing such models is expected.
